# Effects of cadmium exposure during the breeding period on development and reproductive functions in rare minnow (*Gobiocypris rarus*)

**DOI:** 10.3389/fphys.2023.1163168

**Published:** 2023-04-21

**Authors:** Liangxia Su, Huanhuan Li, Ning Qiu, Yinrui Wu, Bing Hu, Rui Wang, Jun Liu, Jianwei Wang

**Affiliations:** ^1^ School of Animal Science and Nutritional Engineering, Wuhan Polytechnic University, Wuhan, China; ^2^ Tianjin Research Institute for Water Transport Engineering, Tianjin, China; ^3^ Fujian Key Laboratory of Special Aquatic Formula Feed, Fuzhou, China; ^4^ The Key Laboratory of Aquatic Biodiversity and Conservation of Chinese Academy of Sciences, Institute of Hydrobiology, Chinese Academy of Sciences, Wuhan, Hubei, China

**Keywords:** parental effect, cadmium, *Gobiocypris rarus*, reproduction capability, toxicological effect

## Abstract

Cadmium is a common reproductive toxin in aquatic systems. Cd exposure of fish species at high concentrations can severely affect the reproductive function of fish. However, the underlying toxicity of cadmium exposure at low concentrations on the reproductive function in parental fish remains unclear. To investigate the impacts of cadmium exposure on reproductive capability, eighty-one male and eighty-one female rare minnows (*Gobiocypris rarus*) were exposed to cadmium at 0 (control group), 5 and 10 μg/L for 28 days, and then transferred into clean water to pair spawn. The results showed that cadmium exposure at 5 or 10 μg/L for 28 days in rare minnows could reduce the success rates of pair spawning in parent rare minnows, lessen no-spawning activities, and prolong the time for first spawning. Furthermore, the mean egg production of the cadmium exposure group increased. The fertility rate of the control group was significantly higher than that of the 5 μg/L cadmium exposure group. Anatomical and histological data further revealed that the intensity of atretic vitellogenic follicles significantly increased and spermatozoa vacuolated after cadmium exposure (*p* < 0.05), but slightly increased the condition factor (CF), and relatively stable gonadosomatic index (GSI) values were also observed in the cadmium exposure groups. These observed results indicated that cadmium exposure at 5 or 10 μg/L affected the reproductive activity of paired rare minnow by accumulating Cd in the gonads, and the effect diminished over time. The reproductive risk of low-dose cadmium exposure to fish species remains a cause for concern.

## 1 Introduction

Cadmium (Cd) is a heavy metal found in the natural environment (Luo X. et al., 2015). Generally, the Cd levels in the drinking water are usually less than 1 μg/L (Anetor, 2012; Zhao et al., 2015). However, the concentration of Cd in the aquatic environment increased with the rapid development of society and industry (Luo Y. et al., 2015; Zheng et al., 2019). For example, the Cd concentration in Longjiang River water ranged from 0.05 to 2.9 μg/L (Zhao et al., 2018). Additionally, the Cd concentration in Hengshi River water was 62.74 μg/L (Liao et al., 2017).

Numerous studies demonstrated that Cd in the aquatic environment can be toxic to aquatic organisms, such as nile tilapia (*Oreochromis niloticus*) (Almeida et al., 2002), palaemonid shrimp (*Palaemon macrodactylus*) (Zhang et al., 2021), and sea urchin (*Paracentrotus lividus*) (Migliaccio et al., 2015). For fish species, Cd exposure may result in their tissue injury (Costa et al., 2013), and in physiological alterations (Kaur et al., 2018; Yu et al., 2021), and may affect their development, growth (Liu et al., 2011), and reproduction (Tilton et al., 2003; Wu et al., 2013). Recent studies indicated that 0.3 mg/L Cd exposure, as a reproductive toxic pollutant, decreased the gonadosomatic index in tilapia (*Orechromis Niloticus*) (Hayati et al., 2020), and a low level of Cd accumulation in semen might contribute to male infertility by reducing the sperm quality of tilapia (Luo X. et al., 2015) and fathead minnows (Sellin and Kolok, 2006a). Moreover, Sellin and Kolok (2006a) found that female fathead minnows (*Pimephales promelas*) exposed to Cd decreased their pair-spawning success, fecundity, fertilization success, hatching success and offspring mortality. These studies demonstrated that Cd exposure resulted in reproductive abnormalities in fish species. However, these studies focused on the effects of Cd exposure on female or male fish species, which may not fully explain the reproductive toxicity of Cd in the natural environment. Therefore, it is necessary to explore the effect of Cd exposure on the reproductive capacity of rare minnow by exposing females and males separately.

Rare minnow, *Gobiocypris rarus* (Re and Fu, 1983), is a native Chinese fish species (Wang and Cao, 2017). Due to its short reproductive cycle and its sensitivity to environmental pollutants, the national standard of China (GB/T29763-2013) has recommended the use of rare minnow for monitoring water environmental pollutants. Recent reports have focused on the toxicity of Cd in rare minnows (Liu et al., 2017; Xiong et al., 2020; Su et al., 2022). However, little information is known about the influence of parental Cd exposure on their reproductive capacity.

In this study, 8-month-old rare minnows (♀:♂ = 1:1) were exposed to 0 (control group), 5 or 10 μg/L of Cd for 28 days (d) and then transferred into clean water to pair spawn. The impacts of Cd exposure on the development and reproductive capacity of parental fish were investigated.

## 2 Materials and methods

### 2.1 Chemical reagents

Cadmium chloride was of analytical grade and purchased from Sinopharm Chemical Reagents Co., Ltd. (Beijing, China). Stock solution (1 g/L Cd^2+^) was prepared with double-deionized water and stored at 4°C. The 5 or 10 μg/L Cd exposure solutions were prepared by adding 25 or 50 μL stock solution to 4.975 or 4.950 L double-deionized water during the experimental period.

### 2.2 Fish rearing

One hundred and sixty-two healthy and mature rare minnow (♀:♂ = 1:1, 8 months old), spawned within 7 days, were collected from the National Aquatic Biological Resource Center, NABRC. These fish were randomly cultured in several 3-L polycarbonate tanks. Females and males were cultured separately. No dead fish were recorded during the 7 days acclimatization period. During the period of acclimatization and experimentation, these fish were fed to satiety twice daily with ozone-disinfected frozen red worms (*Chironomus flaviplumus*) (Yuerle, Tianjin, China). The photoperiod was controlled as a 12:12 h light: dark cycle. The water quality parameters were maintained as follows: water temperature, 25.8°C ± 0.95°C, pH, 8.32 ± 0.21, dissolved oxygen, 8.29 ± 0.52 mg/L, and total hardness, 259.73 ± 40.92 mg/L (CaCO_3_). These parameters were determined by a water quality analyzer (HQ30d, Hach, Loveland, Co., United States) on a daily basis. Water or exposure solution was completely replaced daily during the period of acclimatization and experiment to maintain water quality and Cd concentration. All experiments carried out were approved by the Ethics Committee of the Wuhan Polytechnic University in Hubei Province, China (number WPU-F20210401, approval date 1 April 2021).

### 2.3 Experimental design

To study the effect of Cd exposure on gonadal development, 45 female and 45 male rare minnows were separately divided into three treatment groups with three replicates per group (*n* = 5 fish per replicate). According to the limit of cadmium in Chinese fishery water-quality standards (GB 11607-89) and water-body classification, 5 and 10 μg/L Cd was used as the exposure concentration of this experiment. Generally, females and males were exposed to 0 (control group), 5 (exposure group one), and 10 (exposure group two) μg/L Cd for 28 days separately. The culture and feeding condition were maintained to match those in the accumulation period. At a periodic interval of 7 days, three fish from each group were euthanized using 200 mg/L MS-222 to measure body length and body weight. The ovary or testis was rapidly removed with sharp scissors and weighed. The harvested gonadal tissues were washed with saline (0.9% sodium chloride solution). The left gonadal tissues were freeze-dried for Cd analysis and the right gonadal tissues were fixed in 4% neutral formaldehyde solution.

To study the effect of Cd exposure on reproductive capability, 30 females and 30 males were randomly selected for breeding (*n* = 10 fish per replicate). These fish were transferred into aerated tap water and paired (one female and one male). This experiment consisted of three groups with 10 pairs of rare minnows: group one (control group), parental fish were separately exposed at 0 μg/L Cd^2+^ and then paired; group two, parental fish were separately exposed at 5 μg/L Cd^2+^ and then paired; group three, parental fish were separately exposed at 10 μg/L Cd^2+^ and then paired. The culture and feeding condition were maintained to match those in the accumulation period.

### 2.4 Spawningper formance

According to the results of Wang (1992), the spawning behavior was regarded as parental fish swimming at the top of the water and males chasing females against the wall of the tank. Spawning events were recorded at 22:30 daily to count the amount of spawning and the inter-spawning interval for each pair. The parent fish were removed to a new tank to ensure separation from the eggs. Based on the results of Su et al. (2020), resuming spawning rhythm was defined as a second consecutive inter-spawning interval within 5 days. The next day, the eggs were siphoned into 600 mL cylindrical glass containers [12 cm (diameter); Huaou Industry, Yancheng, China] for collection and parental fish were returned to the original tank for further observation. Fertilized and unfertilized eggs were counted to calculate egg production, while unfertilized eggs were discarded. In addition, the fertility rate of each pair was obtained by the ratio of the gastrula to neurula stages eggs to fertilized eggs (Chang et al., 1995). After hatching, larvae were counted to calculate the hatching rate. The whole reproductive experiment lasted for 28 days.

### 2.5 Gonadal development of parent rare minnow

To assess the effects of Cd exposure on the histological parameter of parental fish, the condition factor (CF) and gonadosomatic index (GSI) were calculated. The histological parameters were calculated as: Condition factor (CF) = (W/L^3^) × 100; Gonadosomatic index (GSI, %) = (W_G_/W) × 100, where W stands for body weight, L stands for body length, and W_G_ stands for gonadal weight.

For the analysis of histological observation, the gonads were sliced. To ensure the quality of the sections, the sections of gonads were obtained within 30 days of fixation. The specific production was as follows: the fixed ovary or testis were washed with 50% ethanol; dehydrated in 75% alcohol for 40 min, 85% alcohol for 40 min, 95% alcohol for 40 min absolute alcohol for 40 min and absolute alcohol for 60 min; and then directly embedded in paraffin, serially sectioned (7 μm) with a rotary microtome, followed by staining with hematoxylin and eosin (H&E). These stained sections were processed by Wuhan Service Bio Technology Co., Ltd. To study the difference of ovary and testis development in Cd unexposed and exposed rare minnows, sections were observed under the Olympus CX33 light microscope (Olympus Corporation, Tokyo, Japan) with a camera (magnification is ×1000).

For morphometric evaluation, the intensity of atretic vitellogenic follicles of female rare minnow and the proportion of spermatozoa vacuolate were explored. The identification of atretic vitellogenic follicles and assessment of its intensity were made according to the method described by Corriero et al. [27]. In brief, a clear fragmentation of zona radiata or absorbed yolk granule was regarded as atretic vitellogenic follicles. For each section of females, random selected 6 digital fields were used to calculate the count the intensities of atretic vitellogenic follicles (magnification is ×400). For each testis sections, 10 digital fields chosen randomly were used to calculate the proportion of spermatozoa vacuolate (magnification is ×400). The morphometric parameter was calculated as: the intensity of atretic vitellogenic follicles (%) = the number of atretic vitellogenic follicles/the number of total late vitellogenesis × 100; the proportion of spermatozoa vacuolate (%) = the number of spermatozoa vacuolate/the number of spermatozoa at late spermatogenesis ×100.

### 2.6 Analyses of Cd concentrition in exposure water and gonadal tissue

The measured concentration of Cd was analyzed using inductively-coupled plasma mass spectrometry (ICP-MS) (PekinElmer NexlON 300X; Waltham, United States). Water and gonadal tissues were digested with 2% nitric acid (HNO_3_) and incubating at 50°C overnight with a microwave digestion system (Multiwave 3000, Anton Paar, Graz, Austria). The specific operation was performed according to the method described by Su et al. (2022). The nominal and measured Cd exposure concentrations was presented in Table 1.

**TABLE 1:** The nominal and measured concentrations of Cd (inductively-coupled plasma mass spectrometry (ICP-MS) method) (*n* = 3).

Item	Nominal concentrations (μg/L)	Measured concentrations (μg/L)
Parental cadmium exposure	0	0.059 ± 0.008
	5	3.804 ± 0.088
	10	7.855 ± 0.911

### 2.7 Statistical analysis

All experimental data were presented as mean ± standard deviation (SD). Normality and assumptions of homogeneity of variance were tested using Kolmogorov–Smirnov and Levene tests, respectively. Because normality and homogeneity of variance assumptions were satisfied, statistical differences in spawning capability and histological parameters between the control and parental Cd exposure group were assessed using one-way analysis of variance followed by Tukey’s HSD comparison test. The statistical analysis was conducted using SPSS 19.0 (IBM, Armonk, NY). A *p*-value < 0.05 was deemed a statistically significant difference. GraphPad Prism 7.0 (GraphPad Software Inc., La Jolla, United States) was used to create the figures and Photoshop CS 6.0 (Adobe, San Jose, CA, United States) was used to edit the figures.

## 3 Results

### 3.1 Cd contents in gonadal tissues

**FIGURE 1:**
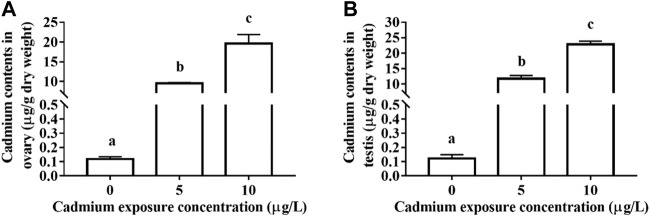
The contents of Cd in the ovary **(A)** and testis **(B)** of rare minnows (Gobiocypris rarus) exposed to 0, 5, and 10 μg/L for 28 days.

A certain amount of Cd is accumulated in the gonads of rare minnow after 28 days Cd exposure. For the control fish, the Cd contents of ovary and testis tissues were 0.119 ± 0.0156 and 0.123 ± 0.0269 μg/g dry weight, respectively. For the ovary of 5 and 10 μg/L Cd exposure fish, the Cd contents were 9.509 ± 0.2210 and 19.638 ± 2.2769 μg/g dry weight, respectively, which was significant higher than those in the control fish (*p* < 0.05, Figure 1). Meanwhile, the Cd contents of testis tissues in Cd exposure groups significantly increased in those control testis (*p* < 0.05, Figure 1).

### 3.2 Effect of cadmium exposure on the spawning activity of parental rare minnow

**FIGURE 2:**
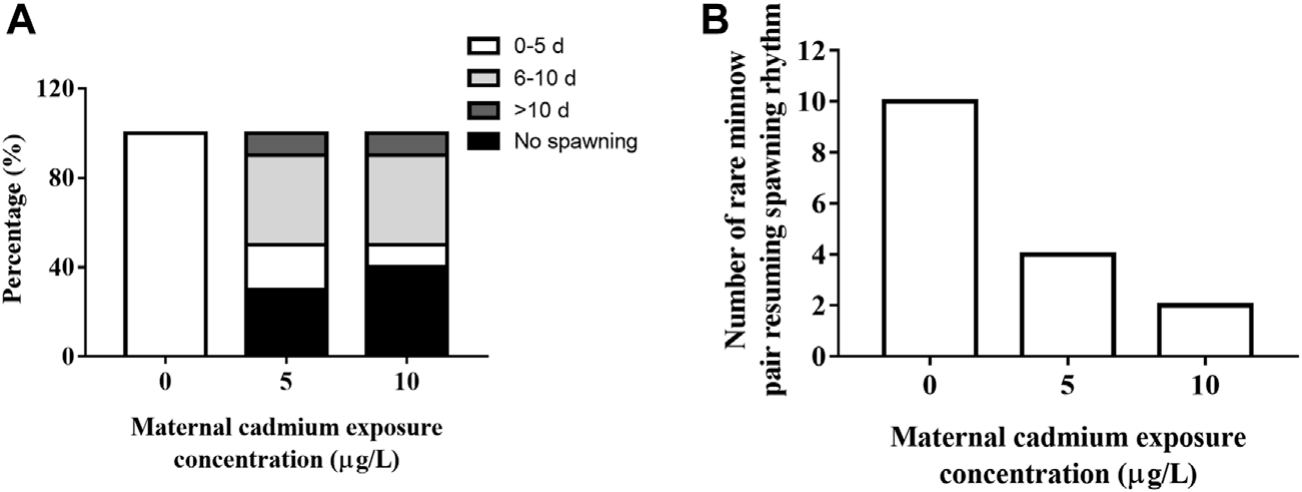
Frequency distribution of first spawning events **(A)** and resuming spawning rhythm **(B)** of rare minnow (*Gobiocypris rarus*) after Cd exposure following a 28 days reproduction period.

Cd exposure severely affected the spawning activity in rare minnow. Success rates of pair-spawning were 100, 70, and 60% in the 0, 5, and 10 μg/L Cd exposure group, respectively. In the 5 and 10 μg/L Cd exposure group, 40% and 10% of pairs spawned between 6 and 10 days and over 10 days, respectively (Figure 2A).

During the reproductive period, paired rare minnow resumed spawning. Compared with the control group, the mean time for first spawning in the 28 days Cd exposure groups significantly increased (Table 2, *p* < 0.05). According to Su et al.’s (2020) criteria for resuming spawning rhythm, all paired rare minnows in the control group spawned within 5 days. However, only four and two pairs of rare minnows in 5 and 10 μg/L Cd exposure resumed the spawning rhythm (Figure 2B). Furthermore, the spawning interval in the 10 μg/L Cd exposure group was 8.19 ± 4.42 days, a significant increase compared to that of the control group (Table 2, *p* < 0.05). The intervals in the 5 μg/L Cd exposure group were not significantly different from the control (Table 2, *p* > 0.05)**.**

**TABLE 2:** Times for first spawning and the average inter-spawning intervals of rare minnows (*Gobiocypris rarus*) after Cd exposure during the 28-day reproduction period.

Cadmium exposure concentration (μg/L)	Time for first spawning (d)	Average inter-spawning interval (d)
0	3.00 ± 1.41^a^	4.50 ± 1.69^a^
5	8.00 ± 5.74^b^	6.41 ± 4.32^ab^
10	8.66 ± 5.35^b^	8.19 ± 4.42^b^

Data are expressed as mean ± SD. Same superscript letters between Cd-exposed group and the control group in the same column are not significantly different (*p* > 0.05).

### 3.3 Cadmium exposure on reproductive performance and reproductive success

Cd exposure influenced the fecundity of paired rare minnows. The mean egg production per spawning increased after Cd exposure, and was significantly higher in the 5 μg/L Cd exposure group than the control group (*p* < 0.05, Table 3). Fertility rates were 46.8% ± 31.9% in the 5 μg/L Cd exposure group, which was significantly lower than those in the control group. However, compared with the control level, the fertility rates in the 10 μg/L Cd exposure group increased (*p* > 0.05, Table 3). Cd exposure fluctuated hatchability, but there were no significant differences (*p* > 0.05, Table 3).

**TABLE 3:** Mean egg production per spawning, mean fertility rate, and mean hatching rate of parental rare minnows (*Gobiocypris rarus*) after Cd exposure following 28 days of reproduction period.

Cadmium exposure concentration (μg/L)	Mean egg production per spawning	Mean fertility rate (%)	Mean hatching rate (%)
0	155.5 ± 101.2^a^	73.1 ± 17.2^a^	87.9 ± 11.6^a^
5	257.6 ± 126.8^b^	46.8 ± 31.9^b^	92.3 ± 6.1^a^
10	233.4 ± 144.8^ab^	80.3 ± 11.8^a^	77.9 ± 31.9^a^

Data are expressed as mean ± SD. Same superscript letters between Cd exposed group and the control group in the same column are not significantly different (*p* > 0.05).

### 3.4 Effect of cadmium exposure on the condition factor of parental fish

**FIGURE 3:**
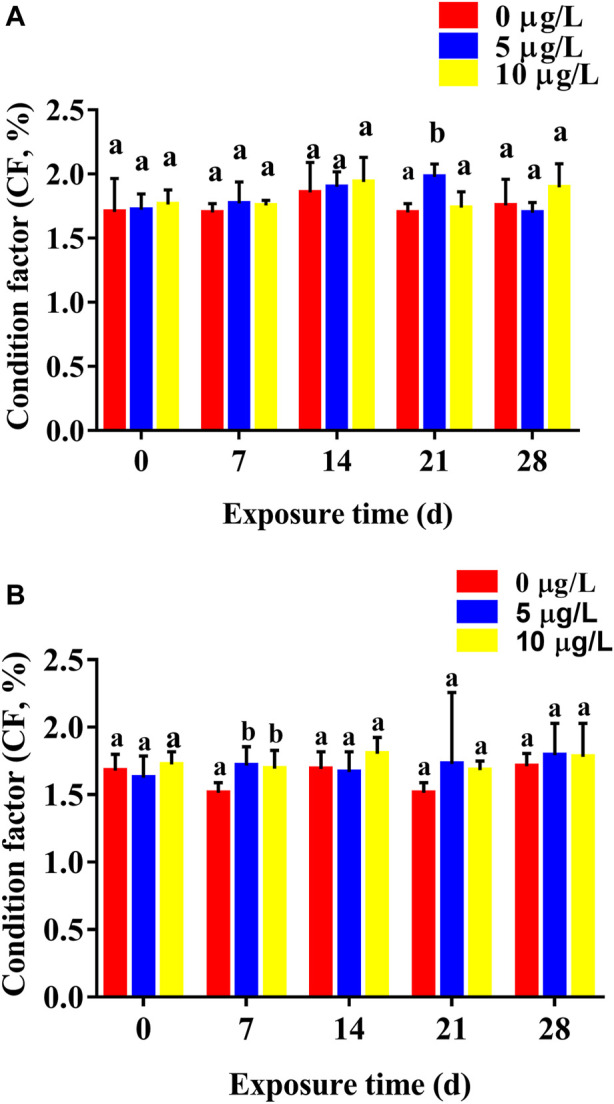
Condition factor (mean ± SD) of rare minnows (*Gobiocypris rarus*) exposed to 0, 5, and 10 μg/L Cd for 28 days (*n* = 3). **(A)** Female rare minnows; **(B)** Male rare minnows. Bar with different letters between the control and exposed groups for the same day indicates statistically significant differences (*p* < 0.05).

Cd exposure increased CF in parental rare minnows, and CF gradually increased with increasing duration of Cd exposure time (Figure 3). For female rare minnows, CF at 21 days of Cd exposure was 1.70% ± 0.07% in the control fish, 1.98% ± 0.10% in the 5 μg/L Cd exposure fish, and 1.74% ± 0.12% in the 10 μg/L Cd exposure fish. Additionally, there was a significantly difference between the 5 μg/L Cd exposure group and the control group (*p* < 0.05) (Figure 3A). For male rare minnows, CF at 7 days of control fish was 1.51% ± 0.07%, which was significantly higher than in the Cd exposure fish (*p* < 0.05) (Figure 3B).

### 3.5 Effect of parental Cd exposure on the gonadosomatic index of parental fish

**FIGURE 4:**
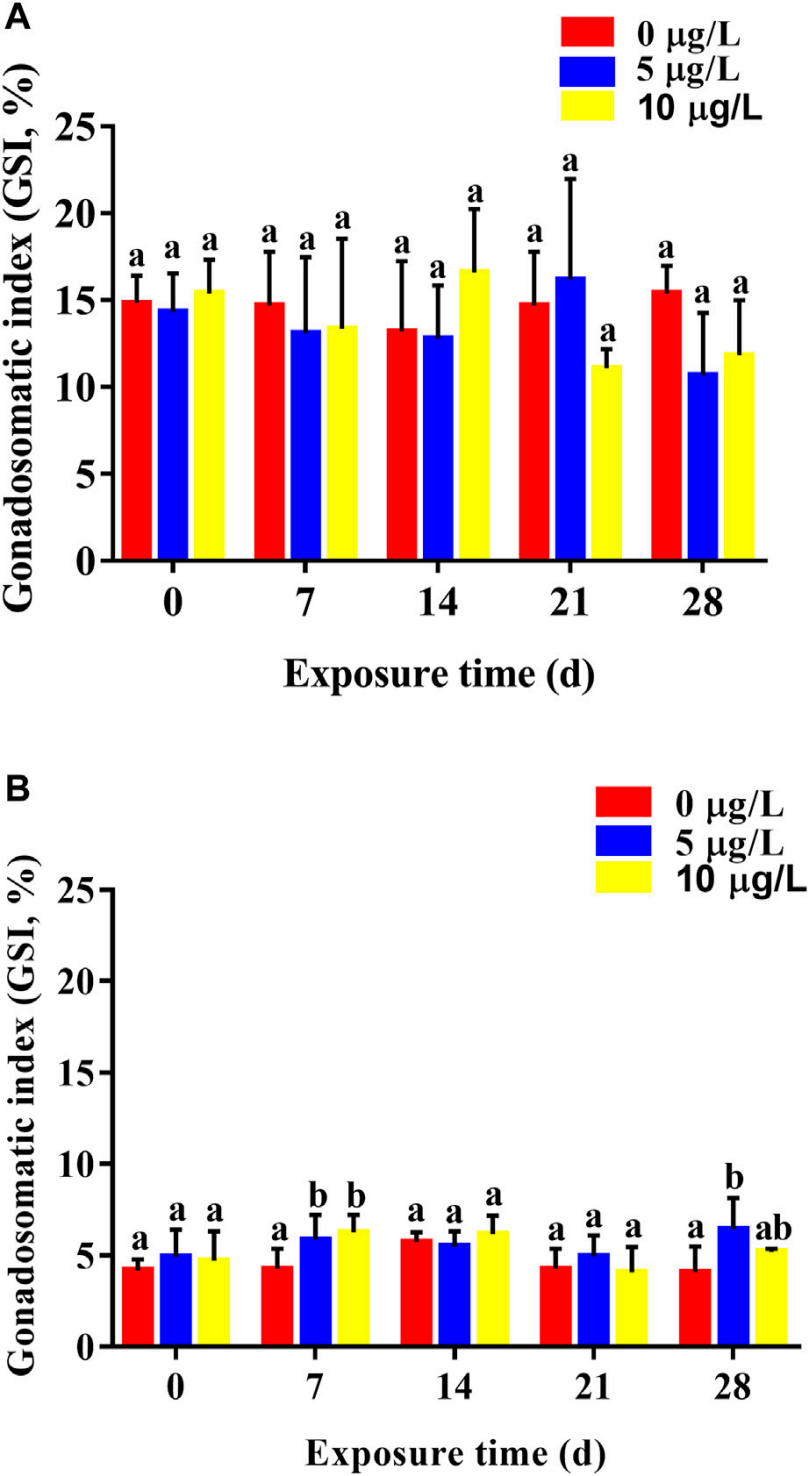
Gonadosomatic index (mean ± SD) of rare minnows (*Gobiocypris rarus*) exposed to 0, 5, and 10 μg/L Cd for 28 days (*n* = 3). **(A)** Female rare minnows; **(B)** Male rare minnows. Bar with different letters between the control and exposed groups in the same day indicates statistically significant differences (*p* < 0.05).

Cd exposure had few effects on GSI values of parental rare minnows. For female rare minnows, GSI values decreased slightly except for females 14 days after exposure to 10 μg/L of Cd and 21 days after exposure to 5 μg/L of Cd. However, there was no significant difference in GSI values between the Cd exposure groups and the control group during the whole Cd exposure period (Figure 4A). For males, GSI values at 7 days Cd exposure were was significantly higher than those in the control group (*p* < 0.05) (Figure 4B). With the duration of Cd exposure, GSI values in the Cd exposure groups showed no significant difference compared with the control levels. However, GSI values at 28 days Cd exposure were 6.42 ± 1.72 in the 5 μg/L Cd exposure group, which was significantly higher than those in the control group (*p* < 0.05) (Figure 4B).

### 3.6 Effect of cadmium exposure on gonadal development

**FIGURE 5:**
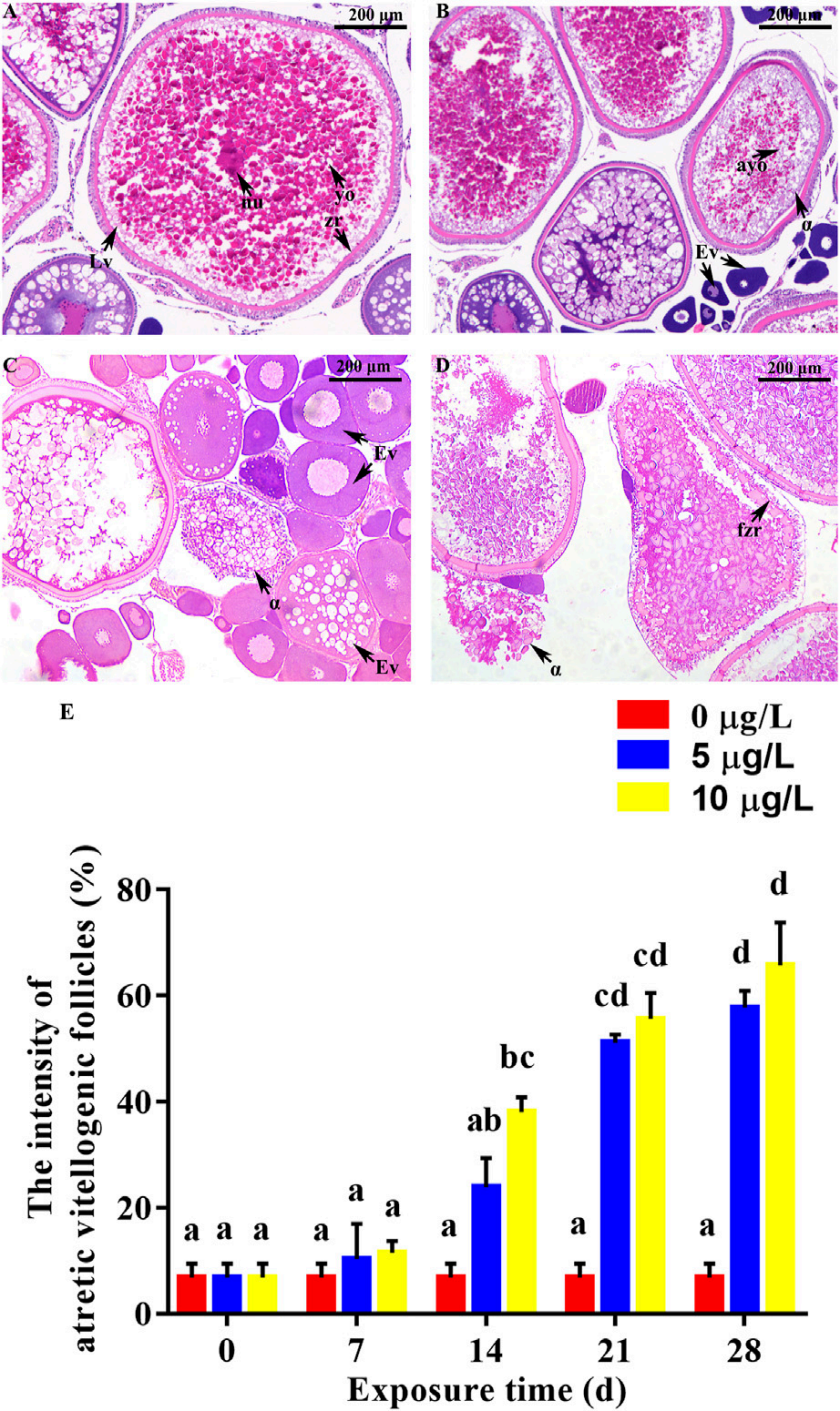
Effect of Cd exposure on ovary tissue in female rare minnows. **(A)** Normal ovary (exposed to 0 μg/L Cd). **(B–D)** Ovary of Cd exposure groups (exposed to 5 or 10 μg/L Cd). **(E)** Intensities of atretic vitellogenic follicles of female rare minnows (*Gobiocypris rarus*). Bar with different letters between the control and exposed groups in the same day indicates statistically significant differences (*p* < 0.05). Lv = oocyte at late vitellogenesis; Ev = oocyte at early vitellogenesis; α = α atretic vitellogenic follicles; yo = yolk granule; nu = nucleus; zr = zona radiata; ayo = absorbed yolk granule; fzr = fragmented zona radiata. α atretic vitellogenic follicle presented in the sections, characterized by absorption of the yolk granule and fragmentation of the zona radiata. Bar with different letters between the control and exposed groups in the same day indicates statistically significant differences (*p* < 0.05).

**FIGURE 6:**
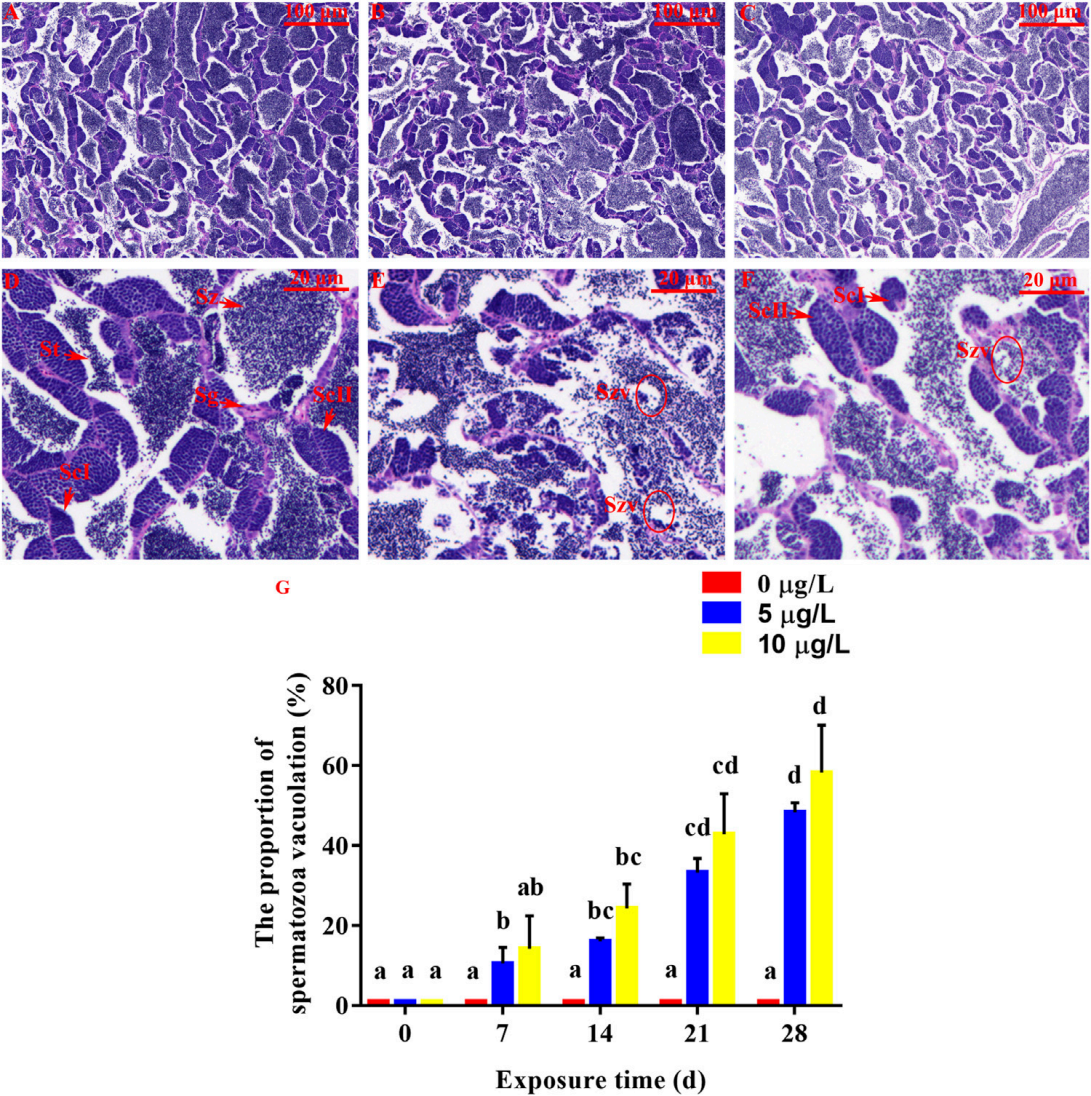
Effect of Cd exposure on testis tissue in rare minnow. **(A, D)** Normal testis (exposed to 0 μg/L Cd). Numerous spermatozoa (Sz) can be observed. **(B, C, E, and F)**. Testis of Cd exposure groups (exposed to 5 or 10 μg/L Cd). Increased spermatozoa vacuolate (Szv) can be observed. **(A–C)** Original picture. **(D–F)** Partial enlarged detail. **(G)** The proportion of spermatozoa vacuolation in male rare minnows (*Gobiocypris rarus*). Bar with different letters between the control and exposed groups in the same day indicates statistically significant differences (*p* < 0.05). Spermatozoa = Sz, spermatogonium = Sg, primary spermatocyte = ScΙ, secondary spermatocyte = ScⅡ, St = spermatids.

Cd exposure adversely affected gonadal development of parental rare minnows (Figures 5, 6). For the control female fish, oocytes in the late vitellogenic stage could be observed, which was characterized by yolk granules and zona radiata (Figure 5A). Moreover, oocytes in the early vitellogenic stage showed small spherical eosinophilic granules or yolk granules in the peripheral ooplasm. Cd exposure resulted in atretic vitellogenic follicles (*α* atresia) in the female parent, which was presented as absorbed yolk granule and fragmented zona radiata (Figures 5B–D). Additionally, the intensity of atretic vitellogenic follicles at 7 days of Cd exposure was 6.85% ± 2.61% in the parental control fish, 10.28% ± 6.68% in the Cd 5 μg/L exposure fish, and 11.52% ± 2.15% in the Cd 10 μg/L exposure fish. There was no significantly difference between the control group and Cd exposure groups (*p* > 0.05, Figure 5E). With the extension of the duration and the increase of concentration of Cd exposure, significant difference in the intensity of atretic vitellogenic follicles was detected between the control group and Cd exposure groups higher in Cd exposure groups than those in the control group (*p* < 0.05, Figure 5E).

For the control male rare minnow, spermatozoa (Sz) were evenly distributed in seminiferous lobules. Additionally, seminiferous lobules contained spermatogonia (Sg), primary spermatocytes (ScΙ), secondary spermatocytes (ScⅡ) and spermatocytes (St) (Figure 6D). The control individuals were found to be at late spermatogenesis, and the lumen of seminiferous lobules was mostly filled with spermatozoa. Spermatozoa vacuolate (Szv) was not observed in the control testis tissues, but parental Cd exposure in male rare minnows led to spermatozoa vacuolate (Figures 6E, F). Additionally, the proportion of spermatozoa vacuolation at 14 days of Cd exposure was 16.03% ± 0.91% in the parental Cd 5 μg/L exposure fish, and 24.29% ± 6.06% in the Cd 10 μg/L exposure fish, which was significantly higher than those in the control fish (*p* < 0.05, Figure 6G). This damage tended to be aggravated the longer duration of exposure concentration and time.

## 4 Discussion

Cd has been recognized as a common toxic pollutant in the aquatic environment (Ahn et al., 2020; Tian et al., 2020). Due to its high carcinogenicity and teratogenicity, Cd has received a great deal of attention. As a result of human concern and governance, the content of Cd in the aquatic environment has been reduced. To fully explain the reproductive toxicity of Cd in the natural environment, female and male rare minnow was exposed to 5 or 10 μg/L Cd solution separately. The present results found that Cd pollution even at low concentrations, could impair the development of parental gonads in rare minnow, affecting their reproductive function.

The present results showed that Cd exposure for 28 days reduced the success rates of pair-spawning in rare minnows and prolonged the time for first spawning. A similar phenomenon was also found in zebrafish (*Danio rerio*) (Wu et al., 2013). Our previous study found that Cd exposure could result in Cd accumulation in their gonads (Su et al., 2022), which was also found in this study. Chouchene et al. (2011) indicated that Cd accumulated in the gonad of zebrafish could hinder its oocyte genesis and result in follicle development disorder. The increased atretic vitellogenic follicle in the late vitellogenic oocytes and spermatozoa vacuolate at late spermatogenesis in this study also confirmed this view. This suggests that accumulation of Cd in the gonads of rare minnow causes no-spawning activities. The fullness of yolk granules is a prerequisite for oocyte maturation, which determines the spawning cycle of the parent fish species to some extent (Su et al., 2020). The histological data showed that Cd exposure resulted in absorbed yolk granules in the present study, which may explain why most of the spawning events in the Cd exposure group occurred in 6–10 days or over 10 days.

The development of fish gonads can directly affect their reproductive capacity, influencing fecundity, fertilization rate, and hatching rate (Young and Woodside, 2001; Su et al., 2012). Sellin and Kolok (2006b) found that female fathead minnows exposed to Cd experienced decreased mean egg production, fertilization rate and hatching rate of offspring. Conversely, the hatching rate of rare minnow offspring whose parents were exposed to 5 or 10 μg/L Cd for 28 days did not decrease, while the mean egg production increased in the present study. This may be due to the relatively low accumulation of Cd in the gonads of rare minnows, which could be gradually removed during the reproductive period. Parents with long spawning intervals will store more nutrients in the gonads, which is conductive to spawning more eggs (Matozzo et al., 2008; Su et al., 2020). The higher CF in the 5 and 10 μg/L Cd exposure group also verified this theory. Thus, the mean egg production of paired rare minnows may be related to the average inter-spawning interval to some extent. In addition, the present results found that the lower fertility rate of rare minnow offspring whose parents were exposed to 5 μg/L Cd for 28 days was significantly lower than those in the 10 μg/L Cd exposure group. This phenomenon may be related to the first time to spawn and average inter-spawning interval. As Su et al. (2020) found in the starvation experiment, parental fish with longer inter-spawning interval spawned more eggs and had a higher fertility rate. Therefore, a longer first spawning time and inter-spawning interval in the 10 μg/L Cd exposure group may be beneficial to the removal of Cd from the gonadal tissue of parental fish, which would increase the fertility rate of parental rare minnow.

The limit of Cd concentration in water is 5 μg/L according to Chinese fishery water quality standards (GB 11607-89). In view of this, little attention has been paid to whether Cd at low concentrations will harm aquatic organisms. However, although the Cd concentration set in this study met the limits of the national fisheries’ water-quality standards, it still could interfere with the reproductive activities of paired rare minnows to some extent. Therefore, the reproductive risk of low-dose Cd exposure to fish species remains a cause for concern.

## 5 Conclusion

In the present study, it was concluded that Cd exposure at 5 or 10 μg/L for 28 days in rare minnows could reduce the success rates of pair-spawning in parent rare minnows, worsened no-spawning activities, and prolonged the time for first spawning. It also caused atretic vitellogenic follicle and vacuolated spermatozoa. Furthermore, the mean egg production of parental Cd exposure group increased. The fertility rate of the control group was significantly higher than that of the 5 μg/L cadmium exposure group. This phenomenon may be due to the reduction of Cd from the gonadal tissue of parental fish with the prolongation of spawning time. The observed results indicated that Cd exposure at 5 or 10 μg/L affected the reproductive activity of paired rare minnow due to the accumulation of Cd in the gonads, although the effects diminished over time.

## Data Availability

The original contributions presented in the study are included in the article/supplementary materials, further inquiries can be directed to the corresponding authors.
